# Clinical characteristics of SARS-CoV-2 infection in children with cystic fibrosis: An international observational study

**DOI:** 10.1016/j.jcf.2020.11.021

**Published:** 2021-01

**Authors:** Robert Bain, Rebecca Cosgriff, Marco Zampoli, Alexander Elbert, Pierre-Régis Burgel, Siobhán B Carr, Claudio Castaños, Carla Colombo, Harriet Corvol, Albert Faro, Christopher H Goss, Hector Gutierrez, Andreas Jung, Nataliya Kashirskaya, Bruce C Marshall, Joel Melo, Pedro Mondejar-Lopez, Isabelle de Monestrol, Lutz Naehrlich, Rita Padoan, Maria Dolores Pastor-Vivero, Samar Rizvi, Marco Salvatore, Luiz Vicente Ribeiro Ferreira da Silva Filho, Keith G Brownlee, Iram J Haq, Malcolm Brodlie

**Affiliations:** a Translational and Clinical Research Institute, Faculty of Medical Sciences, Newcastle University, Newcastle upon Tyne, United Kingdom; b Cystic Fibrosis Trust, London, United Kingdom; c Division of Paediatric Pulmonology, Department of Paediatrics and Child Health, Red Cross War Memorial Children's Hospital, University of Cape Town, South Africa; d Cystic Fibrosis Foundation, Bethesda, MD, United States; e Respiratory Medicine and National Reference CF Center, AP-HP Hôpital Cochin, Paris, France; f Université de Paris, Institut Cochin, Inserm U-1016, Paris, France; g Royal Brompton Hospital and Imperial College London, United Kingdom; h Department of Pulmonology, Hospital de Pediatria JP Garrahan, Buenos Aires, Argentina; i CF Regional Reference Center, Fondazione IRCCS Ca' Granda Ospedale Maggiore Policlinico, University of Milan, Italy; j Sorbonne Université, Inserm, Centre de Recherche Saint-Antoine, Assistance Publique Hôpitaux de Paris (APHP), Hôpital Trousseau, Service de Pneumologie Pédiatrique, Paris, France; k Department of Medicine and Pediatrics, University of Washington, Seattle, WA, United States; l Pediatric Pulmonary and Sleep Medicine, School of Medicine, University of Alabama at Birmingham, Birmingham, AL, United States; m Department of Pulmonology and Children's Research Centre, University Children's Hospital Zurich, Zurich, Switzerland; n Laboratory of Genetic Epidemiology, Research Centre for Medical Genetics, Moscow, Russian Federation; o Instituo Nacional del Tórax, Santiago, Chile; p Pediatric Pulmonology and Cystic Fibrosis Unit, Hospital Clinico Universitario Virgen de la Arrixaca, Murcia, Spain; q Stockholm Cystic Fibrosis Centre Karolinska Institutet, Karolinska University Hospital, Huddinge, Stockholm, Sweden; r Universities of Giessen and Marburg Lung Center, German Center of Lung Research, Justus‐Liebig‐University Giessen, Giessen, Germany; s Pediatric Pulmonology and Cystic Fibrosis Unit, Osakidetza, Hospital Universitario Cruces, Barakado, Bizkaia, Spain; t Pediatric Pulmonology Unit, Instituto da Criança do Hospital das Clínicas da FMUSP, São Paulo, São Paulo, Brazil; u Paediatric Respiratory Medicine, Great North Children's Hospital, Newcastle upon Tyne Hospitals NHS Foundation Trust, Newcastle upon Tyne, United Kingdom; v Cystic Fibrosis Support Center, Department of Paediatric, University of Brescia, Italy; w National Center Rare Diseases, Undiagnosed Rare Diseases Interdepartmental Unit Istituto Superiore di Sanità, Rome, Italy

**Keywords:** COVID-19, Cystic fibrosis, Children

## Abstract

**Background:**

The presence of co-morbidities, including underlying respiratory problems, has been identified as a risk factor for severe COVID-19 disease. Information on the clinical course of SARS-CoV-2 infection in children with cystic fibrosis (CF) is limited, yet vital to provide accurate advice for children with CF, their families, caregivers and clinical teams.

**Methods:**

Cases of SARS-CoV-2 infection in children with CF aged less than 18 years were collated by the CF Registry Global Harmonization Group across 13 countries between 1 February and 7 August 2020.

**Results:**

Data on 105 children were collated and analysed. Median age of cases was ten years (interquartile range 6–15), 54% were male and median percentage predicted forced expiratory volume in one second was 94% (interquartile range 79–104). The majority (71%) of children were managed in the community during their COVID-19 illness. Out of 24 children admitted to hospital, six required supplementary oxygen and two non-invasive ventilation. Around half were prescribed antibiotics, five children received antiviral treatments, four azithromycin and one additional corticosteroids. Children that were hospitalised had lower lung function and reduced body mass index Z-scores. One child died six weeks after testing positive for SARS-CoV-2 following a deterioration that was not attributed to COVID-19 disease.

**Conclusions:**

SARS-CoV-2 infection in children with CF is usually associated with a mild illness in those who do not have pre-existing severe lung disease.

## 1 Introduction

The COVID-19 global pandemic caused by the severe acute respiratory syndrome coronavirus 2 (SARS-CoV-2) has had a profound impact around the world [1], [2]. A spectrum of respiratory disease has been observed ranging from mild (asymptomatic or flu-like symptoms) to severe (acute respiratory distress syndrome, fulminant respiratory failure and high mortality) [2]. Various extrapulmonary manifestations of COVID-19 have also been recognised that include haematological, renal, gastrointestinal, cardiovascular and neuropsychiatric problems [3]. The presence of co-morbidities, including underlying respiratory problems, has been identified as a key risk factor for severe disease along with older age [4], [5], [6]. Current reports suggest that SARS-CoV-2 infection is generally less severe in children than in adults [6], [7]. Most children remain asymptomatic or experience only mild symptoms during infection [5], [6]. Notably, a rare multisystem inflammatory syndrome in association with SARS-CoV-2 infection has been described in a small subset of children and young people [8].

In view of the severity of COVID-19 and high transmissibility of SARS-CoV-2, advice was issued in many countries at the outset of the global pandemic for people with cystic fibrosis (CF) and their families to strictly isolate to reduce the chances of contracting the virus [9], [10]. Respiratory viruses are known to cause pulmonary exacerbations and substantial morbidity in people with CF [11]. There is some evidence to suggest that the innate immune response of CF airway epithelia to respiratory viruses is impaired [12], [13], [14]. During the 2009 influenza A (H1N1) pandemic infection was associated with substantial morbidity in people with CF and a high mortality rate in those admitted to intensive care [15], [16]. Conversely, other aspects of CF airway pathophysiology may mitigate against severe COVID-19 disease [17], [18]. CF airway epithelial cells may be less susceptible to coronavirus infection due to altered intracellular processes involved in host defence and viral replication, for example autophagy, unfolded protein response and NOD-, LRR- and pyrin domain-containing protein 3 inflammasome [17]. SARS-CoV-2 has also been shown to mimic the proteolytic activation of the epithelial sodium channel by furin cleavage [19]. This provides a potential mechanism for reduced entry of SARS-CoV-2 in to CF airway epithelial cells.

Detailed information on the clinical course of COVID-19 in children with CF is lacking and represents an important knowledge gap. There are two individual case reports in children with varying levels of pre-existing lung disease [20], [21]. The first involves a nine-year-old girl with severe CF lung disease who tested positive for SARS-CoV-2 at the same time as her father was hospitalised with COVID-19 [21]. This illness was associated with minor haemoptysis but no other change in her respiratory status [21]. The second case report describes asymptomatic infection in a one-month old infant with CF identified by contact tracing [20]. In May 2020 the CF Registry Global Harmonization Group published an initial report of COVID-19 cases that included a 15 year old child [22]. A range of clinical severity was described in this series with the majority of adult cases being mild or moderate [22]. An updated report including cases up to June 13 2020, was published in November 2020 and recorded 7 deaths [23]. This latest report included 53 children aged <18 years in the dataset but there was no specific analysis of this group.

Increased understanding about SARS-CoV-2 infection in the paediatric age group is crucial to provide accurate and balanced advice that will keep children with CF safe, and recognise the relative risks, but also enable them to lead their lives in the most fulfilling way possible during the ongoing pandemic. This is particularly important given the associated disadvantages and substantial negative effects on quality of life, mental health, schooling and delivery of healthcare of strict isolation measures [8], [24], [25], [26], [27], [28]. Here we detail the clinical course and outcomes of SARS-CoV-2 infection in 105 children with CF collated by an international collaborative group and representing the only large dataset thus far reported.

## 2 Methods

### 2.1 Study design

The ‘Cystic Fibrosis Registry Global Harmonization Group’ is a collaborative international group of patient registries. At the start of the COVID-19 pandemic the group set up fortnightly videoconferences to share experience. Nineteen countries participated in the study that ran between 1 February and 7 August 2020.

Children were defined as individuals aged less than 18 years. Cases were included if they had a confirmed diagnosis of CF and were either diagnosed with SARS-CoV-2 infection via a polymerase chain reaction (PCR) test on a respiratory sample or a clinical diagnosis of COVID-19 was made in a hospital setting. Cases reported via antibody testing alone or self-reporting were excluded.

CF centres that had reported SARS-CoV-2 cases to their registry were asked to complete an anonymised data collection form and a designated country representative then collated all national cases for the study. Representatives provided the most comprehensive data available on each individual case. Data collected included details of the clinical presentation, management and outcome along with relevant patient demographics, including *cystic fibrosis transmembrane conductance regulator* (*CFTR*) genotype, body mass index (BMI), best percentage predicted forced expiratory volume in one second (ppFEV_1_) in the 12 months prior to infection, usual medications, pre-existing CF-related complications, respiratory microbiology and transplant status.

### 2.2 Data analysis

The United Kingdom CF Registry team and the Children's Respiratory Group from Newcastle University performed analyses of pooled cases submitted by participating countries. Data were regarded as missing where specific information was not available at the time of data collection. Results were summarised in tables as numbers and proportions for categorical data and median values with interquartile ranges for continuous data. For categories where some data were missing, the number of cases where data were available was cited in the table. BMI Z-scores adjusted for age and sex were calculated using the World Health Organisation Child Growth Standards [29], [30]. As age was recorded in years, age +0•5 years was used in this calculation for each child. Normality of data was assessed with Shapiro Wilk testing. Subgroup analyses of hospitalised and non-hospitalised cases were performed using either Mann-Whitney test for continuous data or Fisher's exact test for categorical data using GraphPad Prism software (version 8•4•3). *P* values of <0•05 were considered statistically significant.

### 2.3 Information governance and ethics

Each participating country was responsible for ensuring research ethics or institutional approvals were in place for this work as per their individual patient registries.

### 2.4 Role of the funding source

The funding source had no involvement in the collection, analysis or interpretation of data, writing or decision to submit for publication.

## 3 Results

Data on 105 cases of SARS-CoV-2 infection in children with CF were analysed. From the 19 countries that make up the CF Registry Global Harmonization Group, 13 reported cases (Argentina, Brazil, Chile, France, Germany, Italy, Russia, South Africa, Spain, Sweden, Switzerland, United Kingdom and United States of America). Case numbers for individual countries have not been specified to maintain confidentiality.

Details of the method of diagnosis of COVID-19 were available in 96 children. This was via a positive PCR test for SARS-CoV-2 on a respiratory sample in 95 children and by clinical diagnosis of COVID-19 in a hospital setting in one.

### 3.1 Case demographics

Case demographics and features of pre-existing CF disease in the cohort are summarised in Table 1. Median age was ten years and 54% were male. Forty-three (41%) children were homozygous for the F508del *CFTR* mutation. Median best ppFEV_1_ within 12 months prior to infection for those over the age of five years was 94%. Thirty-one had *Pseudomonas aeruginosa* respiratory infection and 31 were on long-term azithromycin treatment. Fifty children were on a CFTR modulator. None were on an active list awaiting transplantation and two children were post-transplant (one lung and one liver).

**Table 1:** Patient demographics and pre-existing cystic fibrosis disease.

Sex *n* = 105	
Male	57 (54%)
Female	48 (46%)
**Age: median years (IQR)***n* = 105	10 (6–15)
0–1 year	11 (10%)
2–4 year	7 (7%)
5–12 year	43 (41%)
13–18 year	44 (42%)
**Body mass index Z-score: median (IQR)***n* = 84	0•08 (−0•81–0•87)
***CFTR* Genotype***n* = 104	
Homozygous F508del	43 (41%)
Heterozygous F508del	32 (31%)
Other	29 (28%)
**Pancreatic insufficient***n* = 100	84 (84%)
**CF related diabetes***n* = 100	9 (9%)
**Lung function***n* = 82	
Best ppFEV1: median (IQR)	94 (79–104)
>70	67 (82%)
40–70	12 (14•5%)
<40	3 (3•5%)
**Respiratory microbiology***n* = 96	
No positive microbiology	20 (21%)
*Staphylococcus aureus*	65 (68%)
*Pseudomonas aeruginosa*	31 (32%)
*Haemophilus influenzae*	10 (10%)
Nontuberculous mycobacteria	7 (7%)
Aspergillus species	1 (1%)
**Allergic bronchopulmonary aspergillosis***n* = 101	8 (8%)
**CFTR modulator therapy***n* = 83	50 (60%)
**Chronic azithromycin treatment***n* = 55	31 (56%)
**Transplant history**	
Solid organ recipient *n* = 105	2 (2%)
Awaiting transplant *n* = 95	0 (0%)
**COVID-19 diagnosis***n* = 96	
Positive respiratory sample PCR	95 (99%)
Clinical diagnosis in hospital setting	1 (1%)

The proportion of each value was calculated from the non-missing data in each group as depicted by n. Abbreviations: CFTR: cystic fibrosis transmembrane conductance regulator; ppFEV1: percent predicted forced expiratory volume in one second; IQR: interquartile range; PCR: polymerase chain reaction.

### 3.2 Symptoms, acute treatments, management and outcome

A summary of the symptomatology, management and treatment of cases is provided in Table 2. Where relevant data were available, just over a quarter of children were asymptomatic. In those with symptoms, fever (73%) and altered cough (72%) were the most common features with 23% experiencing gastrointestinal symptoms. Other less frequently reported symptoms were fatigue, headache and rhinitis each reported in two cases and loss of smell and taste experienced by one child.

**Table 2:** Symptomatology, treatment and management.

Symptoms	
Asymptomatic *n* = 89	26 (29%)
Fever *n* = 63	46 (73%)
Altered cough *n* = 53	38 (72%)
Dyspnoea *n* = 44	13 (30%)
Gastrointestinal *n* = 39	9 (23%)
Myalgia *n* = 37	7 (19%)
**Level of care**	
Community *n* = 82	58 (71%)
Hospital admission *n* = 82	24 (29%)
Intensive care *n* = 83	1 (1%)
**Respiratory support in hospitalised patients**	
New supplemental oxygen *n* = 21	6 (29%)
New non-invasive ventilation *n* = 20	2 (10%)
Invasive ventilation *n* = 20	1 (5%)
**Additional medical management**	
New antibiotic therapy	
- Oral antibiotics *n* = 43	16 (37%)
- Intravenous antibiotics in hospital *n* = 39	14 (36%)
- Intravenous antibiotics at home *n* = 37	4 (11%)
**Experimental antiviral therapy *n*** **=** **36**	5 (14%)
Ritonavir/lopinavir	3 (8%)
Interferon therapy	1 (3%)
Ivermectin	1 (3%)

The proportion of each value was calculated from the non-missing data in each group as depicted by *n*.

Varied levels of care were required with 71% of children being managed in the community and the remainder admitted to hospital. One child was admitted to intensive care six weeks after acute SARS-CoV-2 infection. A range of treatments were used, these included oral (37%) or intravenous (46%) antibiotics with the majority of intravenous antibiotics being administered in hospital. Four children received azithromycin acutely, five antiviral medications and one additional corticosteroids. Of those who were hospitalised, six required new supplementary oxygen therapy, two required non-invasive ventilation and one invasive ventilation. None were supported with extracorporeal membrane oxygenation. No children received experimental treatments for COVID-19 other than azithromycin, interferon or corticosteroids.

At the time of writing, only two cases were described as unresolved, however no formal follow up information is available at this stage. Both solid organ transplant recipients recovered from COVID-19. There were no deaths directly attributed to COVID-19. One child died approximately six weeks after testing positive for SARS-CoV-2. This child had been unwell with deteriorating health during the preceding year including several severe pulmonary exacerbations requiring intensive care admission. During the COVID-19 illness the child was treated with intravenous antibiotics, lopinavir/ritonavir and managed in a ward setting. The child recovered from that acute illness and became negative on repeat SARS-CoV-2 testing. However, six weeks later the child experienced a further severe pulmonary exacerbation requiring admission to intensive care, intubation and mechanical ventilation, and sadly died from respiratory failure.

### 3.3 Characteristics associated with hospitalisation for COVID-19 illness

The characteristics of children admitted to hospital with COVID-19 were compared with those managed in the community in an exploratory analysis using hospitalisation as the best available surrogate of disease severity in our dataset (Table 3). There were no statistically significant differences between sex and *CFTR* genotype in each group. Although the median age was higher in children admitted to hospital, this was not statistically significant. Median BMI Z-score was lower in children admitted to hospital. No pancreatic sufficient children were admitted to hospital with COVID-19. Around half of children with CF-related diabetes were admitted to hospital. Children admitted had a lower median ppFEV_1_ (73%) than those managed in the community (97%). Significantly fewer children were hospitalised who were receiving CFTR modulator therapy, however, it should be noted that access to modulator drugs varied between countries.

**Table 3:** Association of CF disease characteristics and hospitalisation for COVID-19.

	Hospitalised *n* = 24	Not Hospitalised *n* = 58	*P* value
**Sex**			*0•808*†
Male	12 (27%)	32 (73%)	
Female	12 (32%)	26 (68%)	
**Age: median (IQR)**	14 (9–15)	9 (5–15)	0•099*
0–1 year *n* = 9	2 (22%)	7 (78%)	
2–4 year *n* = 6	0 (0%)	6 (100%)	
5–12 year *n* = 29	7 (24%)	22 (76%)	
13–18 year *n* = 38	15 (39%)	23 (61%)	
**Body mass index Z-score: median (IQR)***n* = 61	−0•55 (−1•46- −0•06)	0•32 (−0•55–0•92)	0•015*
**Genotype**			
Homozygous F508del *n* = 36	8 (22%)	28 (78%)	0•222†
Heterozygous F508del *n* = 23	7 (30%)	16 (70%)	>0•999†
Other *n* = 22	9 (41%)	13 (59%)	0•274†
Not known *n* = 1	0 (0%)	1 (2%)	
**Pancreatic status**			*0•029*†
Insufficient *n* = 71	24 (34%)	47 (66%)	
Sufficient *n* = 11	0 (0%)	11 (100%)	
**CF related diabetes**			*0•116*†
Yes *n* = 9	5 (56%)	4 (44%)	
No *n* = 73	19 (26%)	54 (74%)	
**Best ppFEV1 median (IQR)**	73 (48–94)	97 (86–108)	0•002*
>70 *n* = 50	11 (22%)	39 (78%)	
40–70 *n* = 12	8 (67%)	4 (33%)	
<40 *n* = 3	2 (67%)	1 (33%)	
**Allergic bronchopulmonary aspergillosis***n* = 8	5 (63%)	3 (37%)	*-*#
**CFTR modulator therapy**			*0•007*†
No modulator treatment *n* = 30	14 (47%)	16 (53%)	
Modulator treatment *n* = 40	6 (15%)	34 (85%)	

The proportion of each value is calculated from the non-missing data in each group as depicted by *n*.

† Statistical analysis performed using Fisher's exact test.

⁎ statistical analysis performed using Mann-Whitney test.

# no statistical testing performed on allergic bronchopulmonary aspergillosis group due to small numbers. Abbreviations: CFTR: cystic fibrosis transmembrane conductance regulator; ppFEV1: percent predicted forced expiratory volume in one second; IQR: interquartile range.

## 4 Discussion

We describe to our knowledge the first case series of SARS-CoV-2 infection in children with CF. Information was captured on 105 children across 13 countries with a spread of ages ranging from infants through to adolescents. Some of these cases were included within a predominantly adult series, where, crucially, no analysis of the paediatric age group was performed [23]. Specific data on children are urgently required to inform decisions around education for example [28]. The majority of children experienced a relatively mild illness. Over two thirds were managed in the community and of the 24 admitted to hospital six required a period of supplementary low flow oxygen and two needed non-invasive ventilation during their admission. One child was admitted to intensive care, however, this case appears to have been exceptional as discussed below. Two children at the endpoint of data collection were in a recovery phase with the remainder having made a full recovery.

This is a diverse cohort from multiple countries and in-depth comparisons as to how representative the cohort is of children with CF worldwide in general are not possible. The median best ppFEV_1_ in the preceding year was in the normal range at 94%. This level of lung function is comparable with the median ppFEV_1_ for those aged <18 years in 2019 registry reports from the United Kingdom (89.5%) and United States (95%) [31], [32]. Around a third had *P. aeruginosa* infection. This compares to around 25% of children aged <18 years isolating *P. aeruginosa* in the last 12 months in the 2019 United States CF and United Kingdom registries [31], [32]. Due to the international nature of the cohort, availability of CFTR modulator therapy varied between individual countries, with 60% receiving a CFTR modulator.

The distribution of symptoms associated with COVID-19 was similar to those reported in other paediatric, non-CF, cohort studies [5], [6], [7]. Interestingly, just over a quarter were asymptomatic at the time of testing positive for SARS-CoV-2. Many of these children underwent testing as part of the process of an elective admission to hospital during the pandemic or via contact tracing. Although this is unlikely to reflect the true asymptomatic rate, it is in line with other multinational paediatric observational studies [5], [6].

Most children were treated with oral or intravenous antibiotics as is standard practice for any CF respiratory exacerbation [33]. A minority received antiviral medications and none received experimental treatments for COVID-19 other than azithromycin or interferon. This may reflect the generally low severity of acute illness in the cohort. Data showing some clinical benefit from remdesivir in adults with COVID-19 were published in September and October 2020 after the census period of this study [34], [35], [36]. Only one child was recorded as having received additional corticosteroid treatment although the RECOVERY collaborative group only published the results of dexamethasone in hospitalised adult patients in mid-July 2020 and the final date for inclusion of cases in this cohort was August 7 2020 [37].

Some studies have found that those with solid organ transplants are at an increased risk of severe COVID-19 [38]. In our case series, both children who had received solid organ transplants made a full recovery. A small number of adults with CF post-solid organ transplant have died from COVID-19 [23].

We compared the characteristics of children admitted to hospital with those managed in the community as the best available surrogate of COVID-19 disease severity in our dataset. Children admitted to hospital had lower lung function, reduced BMI Z-scores and were less likely to be maintained on a CFTR modulator. No differences in gender were found. Importantly, these comparisons were performed with caution and are exploratory due to the relatively small overall numbers, prevalence of missing data, variable access to CFTR modulators internationally and the important caveat that some children were likely to have been tested for SARS-CoV-2 in association with a hospital admission for other reasons.

There was one clear exception in the cases reported. A child, who had experienced a worrying clinical trajectory over the last year with respect to their underlying CF disease, developed a severe pulmonary exacerbation six weeks after testing positive for SARS-CoV-2. The clinical team involved did not consider this final illness to be related to the SARS-CoV-2 infection or the paediatric multisystem inflammatory syndrome that has been described in temporal association with COVID-19 [8].

This dataset was collated during a global pandemic involving multiple national patient registries and there are inevitable limitations. There are missing values within the dataset partly due to variation in data collection methods between individual registries. This highlights the importance of ongoing work towards registry harmonisation to facilitate and optimise collaborative reporting [39], [40]. Each national registry provided the full data that were available to them about each case. A pragmatic decision was made to not go back to individual clinical teams to request missing data. This would be a hugely time consuming and labour-intensive process across 13 countries and would ultimately delay the publication of urgently required information about the overall clinical course of children with CF who have been infected with SARS-CoV-2. The method of case capture was not exhaustive and was dependant on reporting. Furthermore, at the time of writing formal follow up data were not available. The total paediatric population incorporated within the patient registries involved is estimated to be around 40,000 children. It is likely that the true number of children with CF infected with SARS-CoV-2 is greater than those captured in our study due to differences in testing protocols internationally and variation in false negative rates of COVID-19 tests. Pragmatically, cases were included if children were symptomatic and the clinical team saw signs and symptoms that are characteristically related to COVID-19 [41]. However, only a single case was included on this basis with the remainder having a positive PCR test.

A major strength of this work is that it is the only large detailed case series of COVID-19 specifically focussing on children with CF. This study provides valuable information that will guide children with CF and their families and clinical teams. In summary, our findings suggest that SARS-CoV-2 infection in children with CF is usually associated with a mild illness in those who do not have pre-existing severe lung disease. It remains important, however in our opinion, that children with CF are considered a priority for access to vaccination programmes for SARS-CoV-2 once regulatory approvals are in place. Follow up of children with CF who have experienced infection with SARS-CoV-2 will be important to assess for the development of any long term complications or impact on lung function.

## Declaration of Competing Interest

None related to this work.

P-RB: Not related to this work: reports personal fees from Astra-Zeneca, Boehringer Ingelheim, Chiesi, GSK, Insmed, Novartis, Pfizer, grants and personal fees from Vertex, personal fees from Zambon SBC: Not related to this work: personal fees from Chiesi Pharmaceuticals, Zambon and Insmed and personal fees and non-financial support from Vertex Pharmaceuticals.

CG: Not related to this work: reports grants from CF Foundation, European Commission, NIH, FDA; personal fees from Gilead Sciences (Chair of a Grant Review Committee), Novartis (DSMB Chair for a Clinical Trial); other fees Boehringer Ingelheim (serving as US lead in a phase 2 trial of novel therapy) and Vertex Pharmaceuticals (honoraria for talk at UK LEAD conference).

LN: Not related to this work: reports that he received institutional fees for site participation in clinical trials from Vertex Pharmaceuticals and Boehringer Ingelheim

LVRFSF Not related to this work: reports grants and personal fees from Vertex Pharmaceuticals and Boehringer Ingelheim, grants from Timpel and Diagnostics of America (DASA), personal fees from Roche do Brasil, AbbVie, Sanofi and Glenmark.

MB Not related to this work: investigator-led research grants from Pfizer and Roche Diagnostics; speaker fees paid to Newcastle University from Novartis, Roche Diagnostics and TEVA. Travel expenses to educational meetings Boehringer Ingelheim and Vertex Pharmaceuticals.

RB, RC, MZ, CC, HC, AF, AJ, NK, BM, MDP-V, PM-L, KGB, IJH: none

## Appendix Supplementary materials

Image, application 1
