# Supplementary material for: Clinical characteristics of SARS-CoV-2 infection in children with cystic fibrosis: An international observational study
Source: J Cyst Fibros. 2021 Jan;20(1):25–30. doi: 10.1016/j.jcf.2020.11.021 (PMC7713571; doi:10.1016/j.jcf.2020.11.021)
Supplement: Supplementary file 1 [file mmc1.docx]

# **Supplementary material**

# **Clinical characteristics of COVID-19 in children and young people with cystic fibrosis: an international observational study**

Robert Bain^1^, Rebecca Cosgriff^2^, Marco Zampoli^3^, Alexander Elbert^4^, Pierre-Régis Burgel^5,6^, Siobhán B Carr^7^, Claudio Castaños^8^, Carla Colombo^9^, Harriet Corvol^10^, Albert Faro^4^, Christopher H Goss^11^, Hector Gutierrez^12^, Andreas Jung^13^, Nataliya Kashirskaya^14^, Bruce C Marshall^4^, Joel Melo^15^, Pedro Mondejar-Lopez^16^, Isabelle de Monestrol^17^, Lutz Naehrlich^18^, Maria Dolores Pastor-Vivero^19^, Samar Rizvi^4^, Luiz Vicente Ribeiro Ferreira da Silva Filho^20^, Keith G Brownlee^2^, Iram J Haq^#1,21^ and Malcolm Brodlie^#^*^1,21^

^1^Translational and Clinical Research Institute, Faculty of Medical Sciences, Newcastle University, Newcastle upon Tyne, UK.

^2^Cystic Fibrosis Trust, London, UK.

^3^Division of Paediatric Pulmonology, Department of Paediatrics and Child Health, Red Cross War Memorial Children's Hospital, University of Cape Town, South Africa.

^4^Cystic Fibrosis Foundation, Bethesda, Maryland, USA.

^5^Respiratory Medicine and National Reference CF Center, AP-HP Hôpital Cochin, Paris, France.

^6^Université de Paris, Institut Cochin, Inserm U-1016, Paris, France.

^7^Royal Brompton Hospital and Imperial College London, UK.

^8^Department of Pulmonology, Hospital de Pediatria JP Garrahan, Buenos Aires, Argentina.

^9^CF Regional Reference Center, Fondazione IRCCS Ca' Granda Ospedale Maggiore Policlinico, University of Milan, Italy.

^10^Sorbonne Université, Inserm, Centre de Recherche Saint-Antoine, Assistance Publique Hôpitaux de Paris (APHP), Hôpital Trousseau, Service de Pneumologie Pédiatrique, Paris, France.

^11^Department of Medicine and Pediatrics, University of Washington, Seattle, Washington.

^12^Pediatric Pulmonary and Sleep Medicine, School of Medicine, University of Alabama at Birmingham, Birmingham, Alabama.

^13^Department of Pulmonology and Children’s Research Centre, University Children’s Hospital Zurich, Zurich, Switzerland.

^14^Laboratory of Genetic Epidemiology, Research Centre for Medical Genetics, Moscow, Russian Federation.

^15^Instituo Nacional del Tórax, Santiago, Chile.

# ^16^Pediatric Pulmonology and Cystic Fibrosis Unit, Hospital Clinico Universitario Virgen de la Arrixaca, Murcia, Spain.

^17^Stockholm Cystic Fibrosis Centre Karolinska Institutet, Karolinska University Hospital, Huddinge, Stockholm, Sweden.

^18^Universities of Giessen and Marburg Lung Center, German Center of Lung Research, Justus‐Liebig‐University Giessen, Giessen, Germany.

^19^Cystic Fibrosis Unit, Cruces University Hospital, Bilbao, Spain.

^20^Pediatric Pulmonology Unit, Instituto da Criança do Hospital das Clínicas da FMUSP, São Paulo, São Paulo, Brazil.

^21^Paediatric Respiratory Medicine, Great North Children’s Hospital, Newcastle upon Tyne Hospitals NHS Foundation Trust, Newcastle upon Tyne, UK.

^#^Equal contribution

*Corresponding author: Malcolm Brodlie, MRC Clinician Scientist/Honorary Consultant in Paediatric Respiratory Medicine, Level 3, Clinical Resource Building, Great North Children’s Hospital, Queen Victoria Road, Newcastle upon Tyne, UK, NE1 4LP. E-mail: malcolm.brodlie@ncl.ac.uk. Tel: +44 191 2336161.

We are grateful to the following clinicians who contributed paediatric COVID-19 cases to the ‘Cystic Fibrosis Registry Global Harmonization Group’ via their national coordinator(s) and are collaborating authors.

| **Name** | **Institution** | **Country** |
| --- | --- | --- |
| Constantino G B Cartaxo | Universidade Federal da Paraíba, João Pessoa | Brazil |
| Daniela G Meneses | Universidade Federal de Sergipe, Aracajú | Brazil |
| Véronique Boussaud | Cochin Hospital, Paris | France |
| Graziella Brinchault | Rennes | France |
| Emmanuelle Coirier-Duet | Versailles | France |
| Jean-Christophe Dubus | Marseille | France |
| Dominique Grenet | Foch Hospital, Suresnes | France |
| Sandra de Miranda | Foch Hospital, Suresnes | France |
| Laurence Beaumont | Foch Hospital, Suresnes | France |
| Reem Kanaan | Cochin Hospital, Paris | France |
| Muriel Lauraens | Caen | France |
| Clémence Martin | Cochin Hospital, Paris | France |
| Marie Mittaine | Toulouse | France |
| Anne Prévotat | Lille | France |
| Martine Reynaud-Gaubert | Marseille | France |
| Isabelle Sermet-Gaudelus | Necker Hospital, Paris | France |
| Aurelie Tatopoulos | Nancy | France |
| Raphael Chiron | Montpellier | France |
| Marie-Laure Dalphin | Besancon | France |
| Michele Gerardin | Robert Debre Hospital, Paris | France |
| Laurence Weiss | Strasbourg | France |
| Nathalie Wizla | Lille | France |
| Sophie Ramel | Roscoff | France |
| Rosaria Casciaro | IRCCS Istituto Giannina Gaslini, Genova | Italy |
| Valeria Daccò | Fondazione IRCCS Ca' Granda Ospedale Maggiore Policlinico, Milano | Italy |
| Anna Folino | Azienda Ospedaliera Universitaria, Città della salute e della Scienza, Torino, | Italy |
| Piercarlo Poli | ASST-Spedali Civili, Brescia | Italy |
| Massimo Maschio | IRCCS Materno Infantile Burlo Garofolo, Trieste | Italy |
| Elena Kondratyeva | Research Centre for Medical Genetics | Russia |
| Elena Zhekayte | Research Centre for Medical Genetics | Russia |
| Mariya Mukhina | Morozov State Pediatric Teaching Hospital | Russia |
| Olga Simonova | Federal Academic Centre of Children's Health, Healthcare Ministry of Russia | Russia |
| Carla Els | Linksfiled Mediclinic, Johannesburg | South Africa |
| Isidoro Cortell-Aznar | Hospital Universitario y Politécnico La Fe, Valencia | Spain |
| Alejandro López-Neyra | Hospital Universitario Ramón y Cajal, Madrid | Spain |
| Marta Ruiz de Valbuena | Hospital Universitario La Paz, Madrid | Spain |
| Jordi Costa-Colomer | Hospital Sant Joan de Déu, Barcelona. | Spain |
| Silvia Gartner | Hospital Universitari Vall d´Hebrón, Barcelona. | Spain |
| Marita Gilljam | Respiratory Medicine, Sahlgrenska University Hospital, Gothenburg | Sweden |
| Ulrika Lindberg | Department of Clinical Sciences, Respiratory medicine and Allergology, Lund University; Skane University Hospital, Lund | Sweden |
| Stefanie Diemer | Department of Pediatrics, Lund University; Skane University Hospital, Lund | Sweden |
| Maya Desai | Birmingham Children's Hospital | United Kingdom |
| Elaine Gunn | Cystic Fibrosis Trust | United Kingdom |
| Jeremy Hull | Children’s Hospital, Oxford | United Kingdom |
| Anirban Maitra | Manchester Children’s Hospital | United Kingdom |
| Ghulam Mujtaba | Manchester Children’s Hospital | United Kingdom |
| Christopher O’Brien | Great North Children's Hospital | United Kingdom |
| Claire Onyon | Alexandra Hospital | United Kingdom |
| Kevin Southern | Alder Hey Children's Hospital / University of Liverpool | United Kingdom |
